# A prospective cohort study on the incidence and influencing factors of subsyndromal delirium in ICU patients

**DOI:** 10.3389/fpsyt.2026.1742731

**Published:** 2026-04-28

**Authors:** Ying Li, Jieling Huang, Xiuhong Wang, Yuefei Pan

**Affiliations:** 1 College of Sports Science, Jishou University, Jishou, Hunan, China; 2 Faculty of Health Sciences and Sports Education, Macao Polytechnic University, Macao, China; 3 Intensive Care Unit, Shandong Provincial Third Hospital, Jinan, Shandong, China; 4 Neurocritical Care Unit, Nanjing Gaochun People’s Hospital, Nanjing, Jiangsu, China

**Keywords:** ICU, machine learning, predictive modeling, subdelirium, XGB

## Abstract

**Background:**

This study aims to develop and validate a machine learning-based risk prediction model for subsyndromal delirium (SSD) in ICU patients, while identifying key risk factors.

**Method:**

This study was a prospective study, selecting patients who were hospitalized in the ICU from October 2024 to May 2025. We compared seven machine learning algorithms: Random Forest (RF), Decision Tree (DT), K-Nearest Neighbor (KNN), Logistic Regression (LR), Elastic Network (EN), Extreme Gradient Enhancement (XGB), and Support Vector Machine (SVM).

**Result:**

In our study, the prevalence rate of SSD was 37.158%. The comparative analysis shows that XGB is the best predictive model (AUC = 0.84). Feature importance analysis identified four significant predictive factors: Use of vasoactive drugs (0.412), Monthly household income (0.306), Undergone surgery (0.191) and Number of Medications (0.036).

**Conclusion:**

The prediction model based on XGB has a good effect in identifying the risk of SSD in ICU patients. These findings enable clinicians to stratify high-risk groups and implement timely and targeted intervention measures, effectively reducing the risk of adverse consequences. Future multicenter studies should validate these results in larger cohorts.

## 1 Introduction

Delirium, the most prevalent form of brain dysfunction, is diagnosed according to DSM-5 criteria requiring acute onset, symptom fluctuation, inattention, altered consciousness, and cognitive impairment (e.g., disorientation, memory deficits, or language disturbances) (1). Clinically, some patients exhibit partial but incomplete manifestation of these diagnostic criteria, a condition termed subsyndromal delirium (SSD) (2). SSD refers to a condition where a patient presents with partial delirium symptoms but does not meet the diagnostic criteria for delirium, falling between delirium and non-delirium (3, 4). The incidence of SSD in ICU patients ranges from 13.0% to 33.9%, and there is a 29.1% probability of developing delirium (5, 6). Previous studies have found that delirium is a powerful predictor of cognitive decline (7). A prospective multicenter study found that patients with SSD experienced cognitive decline from admission to 3-month follow-up, and early SSD was associated with cognitive dysfunction (8). The occurrence of SSD can affect the length of hospital stay, post-discharge mortality rate and cognitive function of patients, etc. (9, 10), and in addition, it will also increase the burden on family caregivers and the healthcare system (11, 12). Due to the acute onset and dynamic fluctuating disease characteristics of SSD (13), early identification of risk factors for SSD, timely assessment and intervention, and prevention of its progression to delirium are the key concerns of medical staff (14).

Although previous studies have investigated risk factors for SSD in ICU patients, they predominantly rely on single modeling approaches, particularly logistic regression (4, 15, 16). However, such traditional parametric methods are inherently constrained by rigid assumptions, including linearity between predictors and the log-odds of the outcome, as well as the absence of multicollinearity. Given the highly heterogeneous and dynamic nature of the ICU environment, clinical variables often exhibit complex non-linear relationships and high-order interactions that logistic regression fails to capture without explicit manual specification. Moreover, traditional models are susceptible to outliers and typically necessitate rigorous manual feature selection, which can result in information loss and overfitting when processing high-dimensional, sparse, or noisy data. In contrast, machine learning techniques offer a robust alternative capable of overcoming these limitations (17). Advanced algorithms, such as ensemble methods (e.g., Random Forest, XGBoost), can automatically detect intricate patterns and non-linear dependencies within large-scale datasets without relying on pre-defined distributions. They demonstrate superior robustness in handling missing values and multicollinearity, while providing enhanced predictive accuracy and generalizability (18). Moreover, integrated interpretability tools now allow these “black-box” models to offer clinically actionable insights, facilitating more precise risk stratification and personalized decision-making in critical care. While a study by Japanese researchers utilized a delirium model to predict the probability of syndrome occurrence in the ICU, its specificity was merely 57.1%, rendering it ineffective for identifying SSD among ICU patients (19). Similarly, although Liu (20, 21) developed a model for SSD in ICU patients, their work was restricted to individuals undergoing cardiac or orthopedic surgery, which limits the generalizability and accuracy of SSD outcome evaluation for the broader ICU population. Therefore, there is a critical need to develop a dedicated prediction model for SSD in ICU patients. Such a tool would enable healthcare providers to identify high-risk groups early and improve patient prognosis by mitigating risk factors. Therefore, it is necessary to build an SSD prediction model to help medical staff identify high-risk groups at an early stage and improve patient prognosis by reducing risk factors. Machine learning (ML) algorithms are applicable to all types and sizes of data and have attracted significant attention in the development of patient-centered prediction/prognosis models. These models help optimize treatment plans and promote the monitoring and management of health conditions. In our research, we selected commonly used machine learning models: Logistic Regression (LR), Elastic Network (EN), K-Nearest Neighbor (KNN), Decision Tree (DT), Extreme Gradient Enhancement (XGB), Support Vector Machine (SVM), and Random Forest (RF). In our research, we selected the best model through seven machine learning models to test its clinical applicability in predicting SSD in ICU patients and provide appropriate tools for future SSD management.

## 2 Methods

### 2.1 Study design

This study was conducted in two hospitals located in Shandong and Jiangsu provinces, China, employing a prospective cohort study design.

### 2.2 Methods study population and design

Inclusion criteria: Age ≥18 years old, ICU stay ≥24 hours.

Exclusion criteria: Severe audio-visual dysfunction exists; Suffering from mental illness and taking antipsychotic drugs; There are neurodegenerative diseases, such as Alzheimer’s disease and Parkinson’s disease.

### 2.3 Samples

Sample size was calculated based on the principle that event per variable (EPV) ≥ 10 (22). 17 variables were expected to be included in this study, considering a loss to follow-up rate of 10%-20%, so the minimum sample size for modeling was 340. Since the modeled sample size represents 70% of the total sample, the total sample size is at least 364. A total of 375 responses were collected via paper-based questionnaires. After data cleaning and exclusion of 9 incomplete questionnaires, the valid response rate was 97.6%. The research subjects were patients in the ICU from October 2024 to May 2025.

#### 2.3.1 Measures

##### 2.3.1.1 General patient information

This study independently compiled general data by referring to relevant domestic and foreign literature (12, 16, 23–25). The content of the general data is divided into two parts: general demographic data and disease characteristics, totaling 17 items. The contents include Gender, age, educational level, place of residence, way of living, family income, marital status, per capita family income, history of cerebral infarction, hypertension, diabetes, smoking history, drinking history, number of medications used, application of vasoactive drugs, whether surgery was performed, anemia, mechanical ventilation, etc.

##### 2.3.1.2 Intensive care delirium screening scale

The ICDSC scale is an assessment tool suitable for nurses in intensive care units constructed by Bergeron et al. (26) based on DSM-IV. The Chinese version of ICDSC was translated by Liu (14) et al. It includes 8 items, and the scoring grades are divided into yes and no. No is 0 points, yes is 1 point, and the total score is 8 points. When the total score is 1 to 3, subdelirium is considered (14). The Chinese version of the ICDSC scale has good reliability and validity.

#### 2.3.2 Data collection

Prior to data collection, all researchers underwent standardized training. Clinical data from the first 24 hours of ICU admission were extracted from electronic medical records. Daily ICDSC assessments were conducted at 4 PM, beginning 24 hours post-admission and continuing until ICU discharge, delirium onset, or death. Two researchers independently performed evaluations, with consensus results recorded. Discrepancies were resolved through consultation with the intensive care specialist.

#### 2.3.3 Statistical analysis

To ensure model robustness and generalizability, a two-stage variable selection strategy was employed in this study. First, univariate analysis was conducted to preliminarily evaluate the association between each potential predictor and SSD. Subsequently, variables that showed statistical significance in univariate analysis were entered into multivariate logistic regression for further screening. Only independent predictors identified through this process were included in the final machine learning models.

Statistical analyses were performed using SPSS 27.0. Categorical variables were presented as frequencies and percentages (n, %). Group comparisons were conducted using the chi-square test, and potential risk factors were identified through logistic regression analysis (significance level: P < 0.05).

In machine learning, feature selection is often inherently embedded within the modeling process of specific algorithms. For instance, Random Forest automatically evaluates feature importance during the construction of decision trees. This implies that feature selection is not independent of the model itself, and the mechanisms for feature selection are not universally transferable across different algorithms. Based on this characteristic, we first performed univariate analysis and binary logistic regression for preliminary variable selection. This approach helped mitigate the risk of overfitting due to the limited sample size while maintaining clinical interpretability. After variable selection, the dataset was split into a training set (70%) and a test set (30%). Subsequently, the preselected features were applied to seven different machine learning models: LR, EN, KNN, DT, XGB, SVM, and RF. Detailed information regarding the model development process is provided in **Appendix 1**. For each model, a combination of 5-fold cross-validation and grid search was employed to select the optimal hyperparameters, aiming to maximize accuracy on the training set. This ensured robust performance and facilitated effective prediction and comparison on the test set. All models underwent five repetitions of cross-validation to guarantee their stability and reliability. After comparing the performance of these models, the optimal model was selected based on the area under the receiver operating characteristic curve (AUC). A SHapley Additive exPlanations (SHAP) explainer was then constructed to calculate SHAP values, which represent the contribution of each feature to the model’s predictions. Finally, a SHAP summary plot was generated for the XGBoost model to visualize the impact of individual features on the model output.

## 3 Results

### 3.2 The general characteristics of subsyndromal delirium in ICU patients

A total of 366 critically ill patients in the ICU were included, among whom 216 were male (59.016%) and 150 were female (40.984%). Among these participants, 136 (37.158%) were diagnosed with SSD, while the remaining 230 (62.842%) did not exhibit SSD. Detailed demographic and clinical characteristics are shown in **Table 1**.

**Table 1:** General characteristics of the study participants.

Variable	Total	No	Yes	χ²	P
Gender				0.026	0.871
Male	216	135	81		
Female	150	95	55		
Age(Years)				1.922	0.589
≤60	122	75	47		
61-70	105	68	37		
71-80	87	58	29		
≥81	52	29	23		
Educational background				0.346	0.841
Junior high school and below	318	198	120		
High school	30	20	10		
College or above	18	12	6		
Place of Residence				4.830	0.028*
Rural	249	147	102		
Urban	117	83	34		
Living style				0.059	0.808
Living with family	338	213	125		
Living alone	28	17	11		
Marital status				9.783	0.002*
Non-married	306	203	103		
Married	60	27	33		
Monthly household income(**￥**)				30.718	<0.001**
≥4000	137	98	39		
2001-3999	108	80	28		
≤2000	121	52	69		
Use of vasoactive drugs				51.091	<0.001**
No	131	114	17		
Yes	235	116	119		
Undergone surgery				34.390	<0.001**
No	100	87	13		
Yes	266	143	123		
Smoking history				0.690	0.406
No	196	127	69		
Yes	170	103	67		
Alcohol drinking history				0.312	0.576
No	198	127	71		
Yes	168	103	65		
Number of Medications				16.388	<0.001**
<4 types	62	53	9		
≥4 types	304	177	127		
History of diabetes				0.031	0.859
No	278	174	104		
Yes	88	56	32		
Hypertension history				2.360	0.125
No	172	101	71		
Yes	194	129	65		
Mechanical ventilation				0.393	0.531
No	232	143	89		
Yes	134	87	47		
Anemia				0.835	0.361
No	162	106	56		
Yes	204	124	80		
Old cerebral infarction				1.625	0.202
No	320	205	115		
Yes	46	25	21		

*P<0.05, **P<0.01, P<0.001.

### 3.3 Logistic regression analysis

The incidence of Subsyndromal delirium in ICU patients was taken as the dependent variable (No=0, Yes=1). Based on Place of Residence (Rural =0, Urban=1), Marital status (Married=0, Non-married=1), Monthly household income (≥4000 = 0, 2001-3999 = 1,≤2000 = 2), Use of vasoactive drugs (No=0, Yes=1), Undergone surgery (No=0, Yes=1), Number of Medications (<4 types=0, ≥4 types=1) were taken as independent variables, and logistic regression analysis was conducted. A total of 4 risk factors were identified through analysis (P < 0.05), as shown in **Table 2**.

**Table 2:** Logistic regression analysis.

Risk factor	Reference factor	B	SE	Waldx^2^	P	OR	95%CI
Number of Medications	<4 types						
≥4 types		1.045	0.420	6.195	0.013	2.845	1.249-6.480
Monthly household income(**￥**)	≥4000						
≤2000		1.149	0.314	13.422	<0.001	3.156	1.706-5.836
Use of vasoactive drugs	NO						
Yes		1.850	0.316	34.370	<0.001	6.360	3.427-11.806
Undergone surgery	NO						
Yes		1.460	0.352	17.205	<0.001	4.305	2.160-8.582

### 3.4 Performance evaluation

**Figure 1:**
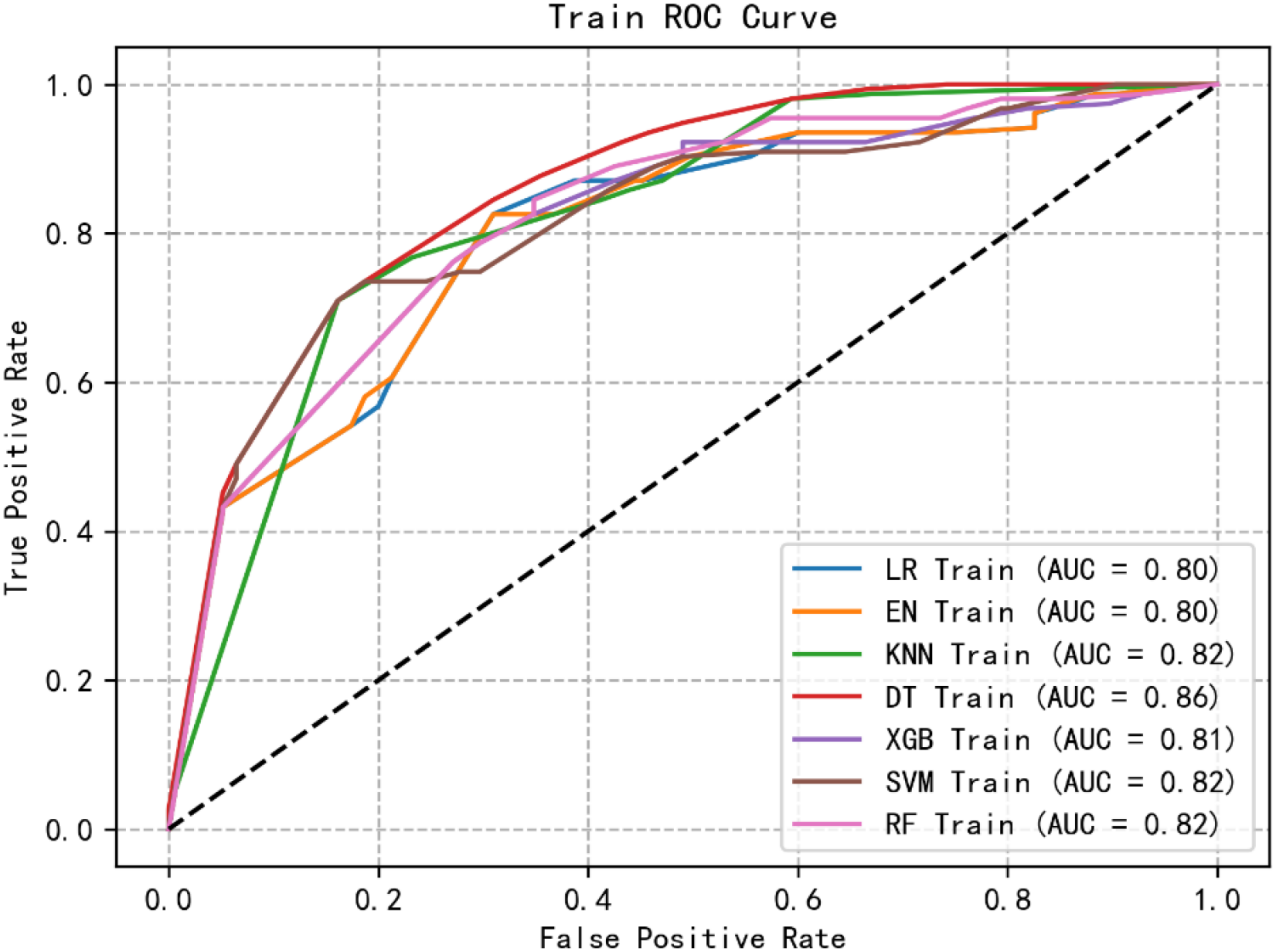
ROC curve of the training group of machine learning algorithms.

**Figure 2:**
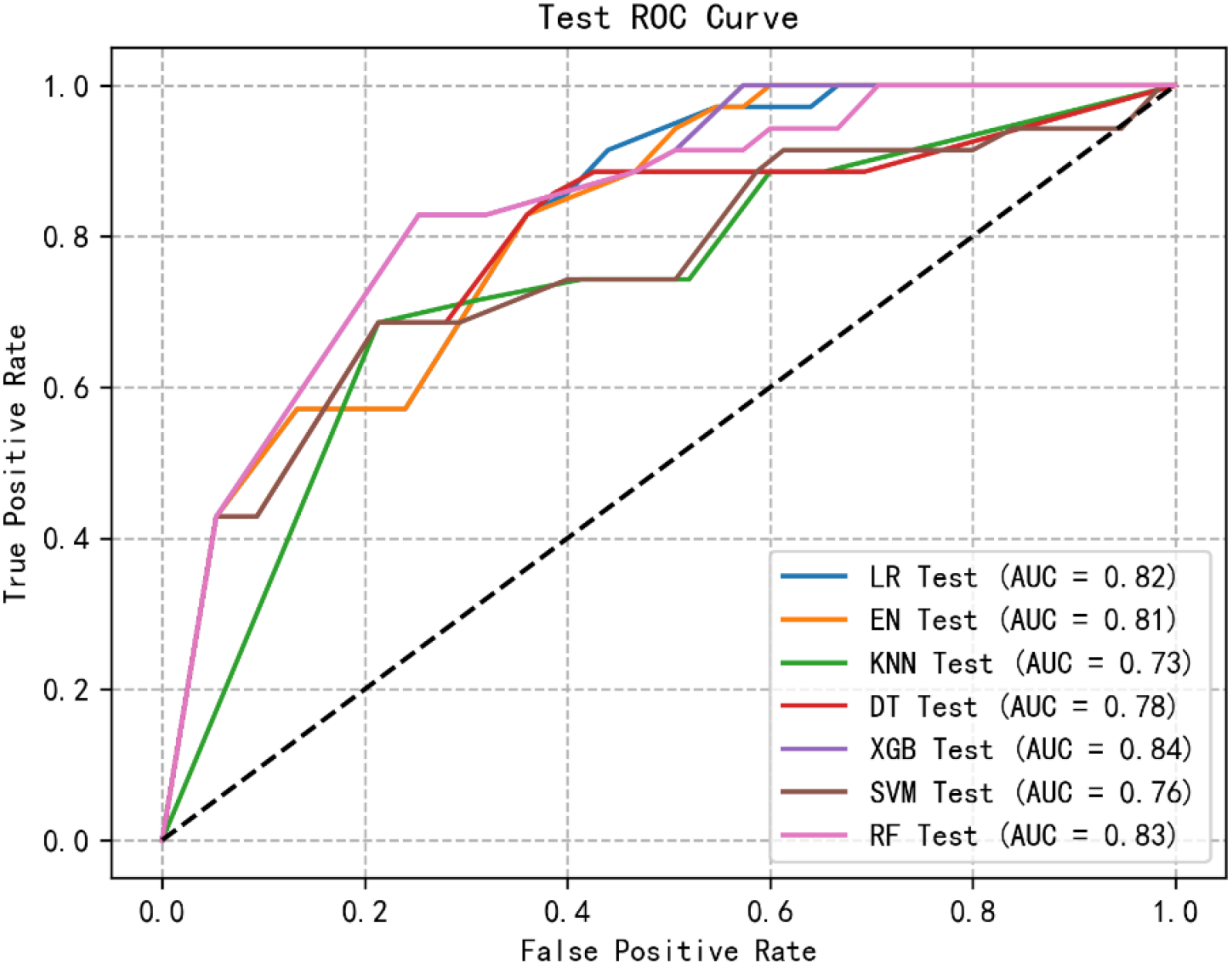
ROC curve of test group of machine learning algorithms.

Through logistic regression analysis, we identified and retained four significant and highly correlated individual characteristics. Using these variables, we constructed seven ML models to predict the risk of Subsyndromal delirium in ICU patients. **Figures 1**, **2** show the receiver operating characteristic (ROC) curves of all models in the test set and validation set, and their predictive performance is quantified by the area under the curve (AUC). Among the test set results, the AUC of the XGB model was the highest (0.84). In addition, we evaluated the accuracy, sensitivity, specificity and confusion matrix of each model (**Table 3**). The XGB model demonstrates the highest accuracy (0.773), and when combined with other performance metrics, it shows the most robust overall performance.

**Table 3:** Comparison of prediction models.

Prediction model	Accuracy	Precision	Recall	F1	Sensitivity	Specificity	Kappa
LR training group	0.758	0.727	0.826	0.773	0.826	0.690	0.516
LR test group	0.700	0.518	0.829	0.637	0.829	0.640	0.404
EN training group	0.758	0.727	0.826	0.773	0.826	0.690	0.516
EN test group	0.700	0.518	0.826	0.637	0.829	0.640	0.404
KNN training group	0.774	0.815	0.710	0.759	0.710	0.839	0.548
KNN text group	0.75 5	0.600	0.686	0.640	0.686	0.787	0.455
DT training group	0.774	0.815	0.710	0.759	0.710	0.839	0.548
DT text group	0.754	0.600	0.686	0.640	0.686	0.787	0.455
XGB training group	0.745	0.738	0.761	0.749	0.761	0.729	0.490
XGB text group	0.773	0.604	0.829	0.699	0.829	0.757	0.523
SVM training group	0.774	0.815	0.710	0.759	0.710	0.839	0.548
SVM text group	0.745	0.600	0.686	0.640	0.686	0.787	0.455
RF training group	0.739	0.703	0.826	0.760	0.826	0.652	0.477
RF text group	0.645	0.470	0.886	0.614	0.886	0.533	0.339

### 3.5 Feature importance ranking

**Figure 3:**
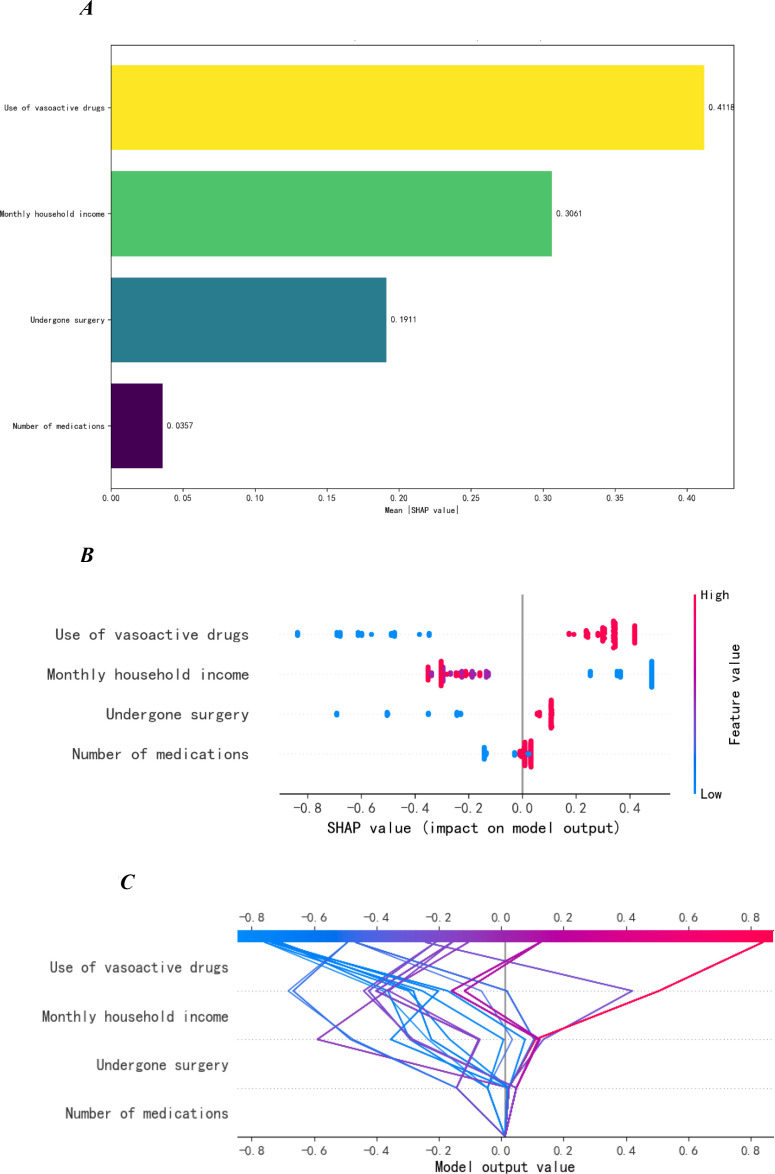
SHAP feature importance plot, SHAP plot and decision plot of the XGBoost model. **(A)** Feature importance plot. **(B)** SHAP plot. **(C)** Decision plot.

The key factors associated with SSD in ICU patients, ranked by feature importance, were: Use of vasoactive drugs (0.412), Monthly household income (0.306), Undergone surgery (0.191), and Number of Medications (0.036), see **Figure 3**.

## 4 Discussion

Seven machine learning algorithms—RF, DT, KNN, LR, EN, XGBoost, and SVM—were employed to develop predictive models for subsyndromal delirium in critically ill ICU patients. Comparative analysis demonstrated that the XGBoost model achieved superior performance across multiple evaluation metrics, including the highest scores in AUC, F1-score, sensitivity, and specificity. These findings suggest XGBoost may represent the optimal algorithm for predicting subclinical delirium in ICU patient populations.

The results of this study revealed 136 cases (37.158%) of SSD patients in the ICU. The results of this study were similar to those of Cheng (27) (34.5%). However it is lower than the research results of Gao (4) (32.0%) and Azuma (19) (31.4%). The consideration may be related to the different survey tools and the population studied (12, 28). At present, the diagnostic criteria for SSD have not been unified. Although SSD and delirium belong to the same disease spectrum, the presentation of two different states of the disease makes the assessment of the two diseases the core issue. However, there is no clear assessment tool for the identification of SSD. Therefore, it is particularly important to explore a targeted assessment tool and an early scientific and effective assessment tool for the identification of SSD in ICU patients, which is crucial for improving the prognosis of SSD in ICU patients.

Our research findings reveal that vasoactive drugs rank first among the risk factors for SSD in ICU patients. The application of vasoactive drugs can cause blood pressure fluctuations in patients, thereby affecting the blood supply to the brain, leading to insufficient oxygen supply to the brain, and thus causing abnormal nervous system function and the occurrence of SSD. Studies have found that the use of phenylephrine can reduce cerebral oxygen saturation, even when the Mean arterial pressure (MAP) significantly increases (29). For critically ill patients using vasoactive drugs, clinical management of blood sugar and drugs should be well carried out, and active measures should be taken to control them. Our findings identified monthly household income as a significant risk factor for SSD in ICU patients. Socioeconomic disparities influence access to healthcare services, with higher-income patients more likely to receive optimal medical care and targeted preventive interventions, thereby reducing the risk of SSD (30). Economic disadvantage has been linked to endocrine-immune dysregulation, chronic stress, and poor mental health, contributing to long-term neuronal, endocrine, and immune system activation (31). This persistent physiological dysfunction may accelerate disease progression, including cardiovascular disorders and cognitive decline—both of which are associated with SSD and delirium development. The number of medications used ≥4 types is a risk factor for SSD in ICU patients. The more severe the condition of ICU patients is, the more drugs are used. There may be interactions among multiple drugs, which can lead to changes in drug metabolism and excretion, thereby increasing the risk of SSD. Therefore, in clinical practice, medical staff need to closely monitor the drug usage of patients, reasonably adjust the drug dosage to reduce the risk of SSD in patients, and take effective intervention measures in a timely manner. The results of this study found that patients who underwent surgery before being admitted to the ICU were more likely to develop SSD. The use of anesthetic drugs during surgery would act on the central nervous system, affecting the synthesis, extraction and secretion of neurotransmitters in the brain (32), thereby causing disorders in the balance of neurotransmitters such as acetylcholine and inducing the occurrence of SSD in patients after surgery (32). Postoperative pain, intraoperative bleeding and intraoperative time can also cause the occurrence of SSD (33, 34).

## 5 Strengths & limitations

This study employed seven machine learning algorithms—RF, DT, KNN, LR, EN, XGBoost, and SVM—to develop a prediction model for SSD in ICU patients. Although XGBoost demonstrated the best predictive performance, several limitations should be noted. The prospective cohort design and moderate sample size may affect the generalizability of the findings. Limited assessment frequency may lead to missed delirium episodes and potentially underestimate the incidence of subsyndromal delirium. Furthermore, the dataset specific to the intensive care unit limits broader applicability, necessitating large-sample multicenter validation studies. Additionally, relying on hospital records to identify psychiatric history may introduce assessment bias, particularly for patients with pre-existing psychiatric disorders. Future studies require methodological improvements in this regard.

## 6 Conclusion

Based on XGB, we predict that the risk factors for SSD in ICU patients include the use of vasoactive drugs, monthly family income, surgical experience and the quantity of medication used. The prediction effect of XGB is relatively good and it has high practical value, which is conducive to screening high-risk groups of SSD. Due to the limitations, it is recommended to further verify the results of this study in the future.

## Data Availability

The original contributions presented in the study are included in the article/**Supplementary Material**. Further inquiries can be directed to the corresponding author.
