# Supplementary material for: A prospective cohort study on the incidence and influencing factors of subsyndromal delirium in ICU patients
Source: Front Psychiatry. 2026 Apr 28;17:1742731. doi: 10.3389/fpsyt.2026.1742731 (PMC13160885; doi:10.3389/fpsyt.2026.1742731)
Supplement: Supplementary file 1 [file Table1.docx]

# Logistic Regression

**Core Formula**

**Linear Combination**

$$z=w^{T}x+b$$

**Sigmoid Function：**

$$P\left( y=1|x \right)=\sigma\left( z \right)=\frac{1}{1+e^{-z}}$$

**Loss Function (Log-Likelihood / Cross-Entropy**)**：**

$$L\left( w \right)=-\sum_{i=1}^{n} \left[ y_{i}\log\left( p_{i} \right)+\left( 1-y_{i} \right)\log\left( 1-p_{i} \right) \right]$$

Logistic regression achieves binary classification by mapping a linear combination of features to a probability space via the Sigmoid function. The model parameters are estimated by maximizing the likelihood function (or equivalently, minimizing the cross-entropy loss).

# 2.Elastic Net

**Core Formula**

**Objective Function：**

$$\min_{w}\left\{ \frac{1}{2n}\sum_{i=1}^{n} \left( y_{i}-x_{i}^{T}w \right)^{2}+\lambda_{1}\left| \right|w\left| \right|_{1}+\lambda_{2}\left| \right|w\left| \right|_{2}^{2} \right\}$$

Elastic Net integrates both L1 (Lasso) and L2 (Ridge) regularization penalties. Specifically, the L1 norm ($\mid\mid w\mid\mid_{1}$) induces sparsity to achieve feature selection, whereas the L2 norm ($\mid\mid w\mid\mid_{2}^{2}$) enhances model stability by handling multicollinearity. Consequently, Elastic Net demonstrates superior performance in high-dimensional datasets or scenarios where features are highly correlated.

# 3.K-Nearest Neighbor, KNN

**Mathematical Formulation**

**Distance Calculation (Euclidean Distance):**

$$d\left( x_{i},x_{j} \right)=\sqrt{\sum_{k=1}^{p} \left( x_{ik}-x_{jk} \right)^{2}}$$

**Classification Decision:**

$$\hat{y}=arg\max_{c}\sum_{i\in N_{k}\left( x \right)} I\left( y_{i}=c \right)$$

K-Nearest Neighbors (KNN) employs an instance-based learning paradigm. For a new query instance, the prediction process involves three steps:

1. Distance Computation: Calculate the Euclidean distance between the query instance and all training samples.
2. Neighbor Selection: Identify the $k$nearest neighbors based on the computed distances.
3. Label Assignment: Determine the class label via a majority voting scheme among the $k$selected neighbors

# 4.Decision Tree

**Mathematical Formulations**

**Gini Index (Gini Impurity):**

$$\boldsymbol{Gini}\left( \boldsymbol{D} \right)\mathbf{=}\boldsymbol{1}\mathbf{-}\sum_{\boldsymbol{k}\mathbf{=}\boldsymbol{1}}^{\boldsymbol{K}} \boldsymbol{p}_{\boldsymbol{k}}^{\boldsymbol{2}}$$

**Information Gain:**

$$Gain\left( D,A \right)=H\left( D \right)-\sum_{v=1}^{V} \frac{\left| D_{v} \right|}{\left| D \right|}H\left( D_{v} \right)$$

**Entropy:**

$$H\left( D \right)=-\sum_{k=1}^{K} p_{k}\log p_{k}$$

Decision trees perform classification by recursively partitioning the feature space. At each node, the feature that maximizes impurity reduction (e.g., measured by Information Gain or Gini Index) is selected as the splitting criterion.

# 5.Random Forest

**Mathematical Formulations**

**Classification Voting Mechanism**

$$\hat{y}=\text{mode}\left( h_{1}\left( x \right),h_{2}\left( x \right),...,h_{T}\left( x \right) \right)$$

**Where:**

- $h_{t}(x)$denotes the prediction of the $t$-th decision tree.
- $T$represents the total number of trees in the forest.

Algorithm Explanation

Random Forest employs a Bagging (Bootstrap Aggregating) ensemble learning paradigm. The construction process involves three key strategies:

Bootstrap Sampling: Multiple training subsets are generated by sampling with replacement from the original dataset.

Feature Randomness: Each decision tree is constructed using a random subset of features at each split node.

Aggregation: The final classification result is obtained through majority voting among all individual trees.

This strategy effectively mitigates the risk of overfitting and enhances the model's generalization performance compared to a single decision tree.

# **6 XGBoost**

**Mathematical Formulation**

**Model Structure:**

$$\hat{y}_{i}=\sum_{k=1}^{K} f_{k}\left( x_{i} \right), f_{k}\in F$$

**Objective Function:**

$$L\left( \phi\right)=\sum_{i=1}^{n} l\left( y_{i},\hat{y}_{i} \right)+\sum_{k=1}^{K} \Omega\left( f_{k} \right)$$

**Regularization Term:**

$$\Omega\left( f \right)=\gamma T+\frac{1}{2}\lambda\left| \right|w\left| \right|^{2}$$

**Algorithm Explanation**

XGBoost belongs to the Gradient Boosting Tree family. Its key features include:

Sequential Learning: The model reduces residuals by sequentially adding trees.

Second-Order Optimization: It utilizes second-order gradient information (Taylor expansion) for more accurate optimization compared to first-order methods.

Regularization: Explicit regularization terms are introduced to control model complexity and prevent overfitting.

Consequently, XGBoost demonstrates high predictive performance across various tasks.

# 7.Support Vector Machine, SVM

### **Mathematical Formulation**

**Optimization Problem (Primal Form):**

$$\min_{w,b,\xi}\frac{1}{2}\left| \right|w\left| \right|^{2}+C\sum_{i=1}^{n} \xi_{i}$$

**Constraints:**

$$y_{i}\left( w^{T}x_{i}+b \right)\geq1-\xi_{i}$$

**Decision Function:**

$$f\left( x \right)=sign\left( w^{T}x+b \right)$$

**Kernel Function Formulation:**

$$f\left( x \right)=sign\left( \sum_{i=1}^{n} \alpha_{i}y_{i}K\left( x_{i},x \right)+b \right)$$

**Algorithm Explanation**

The primary objective of SVM is to find the maximum margin hyperplane that separates different classes.

Maximum Margin: By maximizing the distance between the hyperplane and the nearest data points (support vectors).

Kernel Trick: By employing a kernel function $K(x_{i},x)$, the data is implicitly mapped to a high-dimensional feature space, enabling the solution of non-linear classification problems without explicitly computing the transformation.
